# Inhibition of Shedding of Low‐Density Lipoprotein Receptor–Related Protein 1 Reverses Cartilage Matrix Degradation in Osteoarthritis

**DOI:** 10.1002/art.40080

**Published:** 2017-04-28

**Authors:** Kazuhiro Yamamoto, Salvatore Santamaria, Kenneth A. Botkjaer, Jayesh Dudhia, Linda Troeberg, Yoshifumi Itoh, Gillian Murphy, Hideaki Nagase

**Affiliations:** ^1^ University of Oxford Oxford UK; ^2^ University of Cambridge Cambridge UK; ^3^ Royal Veterinary College Hertfordshire UK

## Abstract

**Objective:**

The aggrecanase ADAMTS‐5 and the collagenase matrix metalloproteinase 13 (MMP‐13) are constitutively secreted by chondrocytes in normal cartilage, but rapidly endocytosed via the cell surface endocytic receptor low‐density lipoprotein receptor–related protein 1 (LRP‐1) and subsequently degraded. This endocytic system is impaired in osteoarthritic (OA) cartilage due to increased ectodomain shedding of LRP‐1. The aim of this study was to identify the LRP‐1 sheddase(s) in human cartilage and to test whether inhibition of LRP‐1 shedding prevents cartilage degradation in OA.

**Methods:**

Cell‐associated LRP‐1 and soluble LRP‐1 (sLRP‐1) released from human cartilage explants and chondrocytes were measured by Western blot analysis. LRP‐1 sheddases were identified by proteinase inhibitor profiling and gene silencing with small interfering RNAs. Specific monoclonal antibodies were used to selectively inhibit the sheddases. Degradation of aggrecan and collagen in human OA cartilage was measured by Western blot analysis using an antibody against an aggrecan neoepitope and a hydroxyproline assay, respectively.

**Results:**

Shedding of LRP‐1 was increased in OA cartilage compared with normal tissue. Shed sLRP‐1 bound to ADAMTS‐5 and MMP‐13 and prevented their endocytosis without interfering with their proteolytic activities. Two membrane‐bound metalloproteinases, ADAM‐17 and MMP‐14, were identified as the LRP‐1 sheddases in cartilage. Inhibition of their activities restored the endocytic capacity of chondrocytes and reduced degradation of aggrecan and collagen in OA cartilage.

**Conclusion:**

Shedding of LRP‐1 is a key link to OA progression. Local inhibition of LRP‐1 sheddase activities of ADAM‐17 and MMP‐14 is a unique way to reverse matrix degradation in OA cartilage and could be effective as a therapeutic approach.

Osteoarthritis (OA) is the most prevalent age‐related joint disorder, but there is no disease‐modifying treatment available except for joint replacement surgery 1. The main cause of the disease is degradation of articular cartilage due to elevated activities of matrix metalloproteinases (MMPs) and ADAMTS. While both ADAMTS‐4 and ADAMTS‐5 have been considered to participate in aggrecan degradation in human OA 2, 3, recent studies by Larkin et al with neutralizing monoclonal antibodies have shown that ADAMTS‐5 is more effective than ADAMTS‐4 in aggrecan degradation in human OA cartilage and nonhuman primates in vivo 4. Collagen fibrils are mainly degraded by collagenolytic MMPs, and MMP‐13 is considered to be the major collagenase in OA cartilage 5, 6, 7.

We have recently found that both ADAMTS‐5 and MMP‐13 are constitutively produced in healthy human cartilage, but they are rapidly taken up by the chondrocytes via the endocytic receptor low‐density lipoprotein receptor–related protein 1 (LRP‐1) and degraded intracellularly 8, 9, 10. These findings suggest that they probably function for a very short period of time to maintain normal homeostatic turnover of extracellular matrix (ECM) components of the tissue. Other proteins that are endocytosed by LRP‐1 include ADAMTS‐4 11 and tissue inhibitor of metalloproteinases 3 (TIMP‐3) 12, 13, indicating that LRP‐1 is a key modulator of cartilage matrix degradation systems. This endocytic pathway is impaired in OA cartilage because of the reduction of protein levels of LRP‐1 in chondrocytes without any significant changes in the level of messenger RNA (mRNA) for LRP‐1, resulting in increased extracellular activity of ADAMTS‐5 8. We thus proposed that the loss of LRP‐1 in OA cartilage is due to proteolytic shedding of the receptor, and that this process shifts normal homeostatic conditions of cartilage to a more catabolic environment, leading to the development of OA.

The aim of this study was to identify the “sheddase” activities that cleave LRP‐1 and release the soluble form of LRP‐1 (sLRP‐1) in human cartilage. We also aimed to test whether inhibition of the sheddase(s) prevents the degradation of cartilage in OA.

## MATERIALS AND METHODS

### Reagents and antibodies

The sources of materials used were as follows: mouse monoclonal anti–LRP‐1 α‐chain antibody (8G1), mouse anti–LRP‐1 β‐chain monoclonal antibody (5A6) that recognizes the ectodomain, BC‐3 mouse monoclonal antibody that recognizes the N‐terminal ^374^ARGSV aggrecan core protein fragments generated by aggrecanase, rabbit anti–ADAM‐10 polyclonal antibody (ab1997), rabbit anti–ADAM‐17 polyclonal antibody (ab2051), and rabbit anti–MMP‐14 monoclonal antibody (ab51074) were from Abcam; mouse anti–FLAG M2 monoclonal antibody, chondroitinase ABC, endo‐β‐galactosidase, bovine nasal septum type II collagen, E‐64, and 4‐(2‐aminoethyl)benzenesulfonyl fluoride (AEBSF) were from Sigma; human recombinant interleukin‐1α (IL‐1α) and tumor necrosis factor (TNF) were from PeproTech; rabbit antitubulin polyclonal antibody (no. 2148) was from Cell Signaling Technology; goat antiactin polyclonal antibody (I‐19) was from Santa Cruz Biotechnology; human plasma IgG (1‐001‐A) was from R&D Systems; solubilized and purified full‐length human LRP‐1 was from BioMac; and a hydroxamate‐based MMP inhibitor, CT1746, was from UCB Celltech.

Anti‐human ADAMTS‐5 catalytic domain rabbit polyclonal antibody was raised in rabbits and characterized 14. Inhibitory monoclonal antibodies against human ADAM‐17 (D1A12) 15, MMP‐14 (E2C6), desmin (negative control antibody) 16, and ADAMTS‐5 (2D3) 9; bovine nasal aggrecan 17, receptor‐associated protein (RAP) 8, recombinant human ADAMTS‐5 lacking the C‐terminus thrombospondin domain with a FLAG tag at the C‐terminus 14, MMP‐13 with a FLAG tag between the signal and propeptide 18, TIMP‐1 19, TIMP‐2 20, TIMP‐3 21, and N‐terminal domain of human TIMP‐3 22 were prepared as described previously. All other reagents used were of the highest available analytic grade.

### Human cartilage tissue preparation and isolation of chondrocytes

Cartilage from femoral condyles of human knee joints was used. Healthy normal articular cartilage was obtained from patients following knee amputation due to soft tissue sarcoma or osteosarcoma with no involvement of the cartilage. Tissue specimens were obtained from 9 patients (6 males, ages 9–57 years, mean age 35.5 years; 3 females, ages 13–19 years, mean age 15.7 years). Human OA articular cartilage was obtained from patients following total knee replacement surgery. Tissues were obtained from 16 patients (8 males, ages 51–86 years, mean age 75.1 years; 8 females, ages 50–82 years, mean age 68.1 years). Dissected cartilage (∼18 mm^3^, ∼20 mg wet weight/piece) was placed in one well of a round‐bottomed 96‐well plate and allowed to rest for 24 hours in 200 µl of Dulbecco's modified Eagle's medium (DMEM) containing 10% fetal calf serum (FCS) before use. The medium was replaced, and the cartilage was rested for a further 24–96 hours in 200 µl of DMEM at 37°C before the assays were performed. Chondrocytes were isolated as described previously 12. Primary chondrocytes were used in the experiments to compare normal and OA chondrocytes, and passaged cells were used in the experiments to identify the LRP‐1 sheddase.

### Western blot analysis of LRP‐1 in cartilage

To analyze LRP‐1 in the cartilage, medium was removed after incubation for various periods of time, and then total protein was extracted by adding 50 µl of 4× sodium dodecyl sulfate (SDS) sampling buffer (200 m*M* Tris HCl [pH 6.8]/8% SDS and 20% glycerol) to each explant in a 96‐well plate. After 1 hour of incubation, the sample buffers were pooled from each condition (3 explants per condition), and 10 µl of samples was analyzed by SDS–polyacrylamide gel electrophoresis (PAGE) under nonreducing conditions and Western blotting using anti–LRP‐1 α‐chain, anti–LRP‐1 β‐chain, and antitubulin antibodies. Immune signals of LRP‐1 and tubulin were quantified using ImageJ software (National Institutes of Health), and the relative amounts of LRP‐1 α‐ and β‐chains in the cartilage extracts were estimated using tubulin as an internal control.

### Immunofluorescence staining of LRP‐1

OA and normal cartilage samples (n = 3 each) were rested in culture with DMEM for 2 days. Explants were then snap‐frozen and sectioned (5‐µm sections) using a CM1900 cryostat (Leica Microsystems). Each sample was fixed with methanol and incubated with anti–LRP‐1 β‐chain antibody for 3 hours at room temperature. Incubation with Alexa Fluor 568–conjugated anti‐mouse IgG (Molecular Probes) for 1 hour at room temperature was used to visualize the antigen signals. Nuclei were stained with DAPI. Samples were viewed using an Eclipse TE2000‐U confocal laser scanning microscope (Nikon). Data were collated using Volocity software (Improvision).

### Quantitative reverse transcriptase–polymerase chain reaction (qRT‐PCR)

Quantitative RT‐PCR was carried out as described previously 8. Briefly, RNA was extracted and isolated from 50 mg of ground cartilage tissue using an RNeasy kit (Qiagen), and complementary DNA was then generated using a reverse transcription kit following the guidelines of the manufacturer (Applied Biosystems). Complementary DNA was then used for real‐time PCR assays using TaqMan technology. The ΔΔC_t_ method of relative quantitation was used to calculate relative mRNA levels for each transcript examined. The 60S acidic ribosomal protein P0 (RPLP0) gene was used to normalize the data. Predeveloped primer/probe sets for LRP‐1, ADAM‐12, and RPLP0 were purchased from Applied Biosystems.

### Western blot analysis of cellular LRP‐1 and sLRP‐1

Chondrocytes (5 × 10^4^) were cultured in 12‐well plates in 2 ml of DMEM containing 10% FCS for 2 days. Cells were rested in 1 ml of DMEM for 24 hours, and the medium was replaced with 1 ml of DMEM and used in the experiments. After incubation for various periods of time, the medium was collected and concentrated 20‐fold using spin filters (Microcon YM‐30; Merck Millipore), and 20 µl of 4× SDS sampling buffer was added to 50 µl of each concentrated medium. The cells were lysed with 200 µl of 2× SDS sampling buffer, and 10 µl of samples were analyzed by SDS‐PAGE under nonreducing conditions and Western blotting using anti–LRP‐1 α‐chain, anti–LRP‐1 β‐chain, and antitubulin antibodies. Immune signals of LRP‐1 and tubulin were quantified using ImageJ software, and the relative amounts of LRP‐1 α‐chain in the medium and LRP‐1 α‐ and β‐chains in the cell lysate were estimated within the linear range of measurements (see Supplementary Figures 1A and B, available on the *Arthritis & Rheumatology* web site at http://onlinelibrary.wiley.com/doi/10.1002/art.40080/abstract) and normalized using tubulin, and those in the standard cell lysates of chondrocytes were used as internal controls. An absolute number of LRP‐1 molecules released into medium was estimated by comparing various concentrations of purified LRP‐1 within a reasonable linear range.

### Flow cytometric analysis of LRP‐1

Cells were plated in 6‐well plates in DMEM containing 10% FCS and incubated until 80% confluent. Cells were rested in 2 ml of DMEM for 1 day, detached using a cell scraper, and fixed for 5 minutes at 4°C with ice‐cold methanol followed by 2 washes with fluorescence‐activated cell sorting (FACS) buffer (phosphate buffered saline containing 5% goat serum and 3% bovine serum albumin [BSA]). Cells were stained for 30 minutes at 25°C with anti–LRP‐1 β‐chain antibody, washed with FACS buffer, and then further incubated with allophycocyanin‐conjugated goat anti‐mouse IgG (BD PharMingen) and isotype control in FACS buffer for 20 minutes at 25°C. Cells were then washed with FACS buffer and analyzed using an LSRII flow cytometer (BD Biosciences), and postacquisition data analysis was performed using FlowJo software version 7.6.1 (Tree Star).

### Analysis of endocytosis of ADAMTS‐5 and MMP‐13

Cells (5 × 10^4^) cultured in 24‐well plates were rested in 500 μl of DMEM for 1 day. The medium was replaced with 500 μl of fresh DMEM with 10 n*M* of ADAMTS‐5 or MMP‐13 in the absence or presence of 2 n*M* or 10 n*M* sLRP‐1 or 500 n*M* RAP at 37°C. After incubation for 0–4 hours, media were collected and the protein was precipitated with 5% trichloroacetic acid and dissolved in 50 μl of 1× SDS sampling buffer containing 5% 2‐mercaptoethanol. All samples were analyzed by SDS‐PAGE under reducing conditions and Western blotting using anti–FLAG M2 antibody or anti–ADAMTS‐5 antibody, respectively. Immune signals for exogenously added ADAMTS‐5 and MMP‐13 detected in the medium were quantified using ImageJ software within the linear range of the measurements (see Supplementary Figures 1C and D, http://onlinelibrary.wiley.com/doi/10.1002/art.40080/abstract), and the amount of each recombinant protein remaining in the medium at each time point was calculated as a percentage of the amount of each recombinant protein at 0 hours.

### Analysis of aggrecanolytic activity of ADAMTS‐5

Purified ADAMTS‐5 (5 n*M*) was preincubated with 0–25 n*M* purified sLRP‐1 in TNCB buffer (50 m*M* Tris HCl [pH 7.5]/150 m*M* NaCl/10 m*M* CaCl_2_/0.01% BSA) containing 0.01% Brij‐35 for 10 minutes at 25°C. The mixture was diluted 100‐fold and incubated with 0.5 mg/ml purified bovine aggrecan for 0–4 hours at 37°C. The samples were deglycosylated as described previously 23. Briefly, aggrecan was deglycosylated in sodium acetate buffer with chondroitinase ABC and endo‐β‐galactosidase (each 0.01 unit/100 µg of aggrecan) for 24 hours at 37°C. Aggrecan was then precipitated using ice‐cold acetone and analyzed by Western blotting using the antibody BC‐3, which recognizes the N‐terminal ^374^ARGSV aggrecan core protein fragments generated by aggrecanase.

### Small interfering RNA (siRNA)–mediated knockdown of membrane‐bound metalloproteinases

Small interfering RNA oligonucleotides for ADAMs and MMP‐14 (On‐TargetPlus SMARTpool siRNA) and nontargeting oligonucleotide were purchased from Thermo Scientific Dharmacon. Cells were plated at a density of 4 × 10^4^ cells/well (12‐well plate) in DMEM containing 10% FCS and incubated until 50% confluent. INTERFERin (PeqLab) was used to transfect cells with siRNA at a final concentration of 20 n*M* in Opti‐MEM I (Gibco). After 48 hours of incubation, the medium was replaced with fresh DMEM with or without 10 ng/ml IL‐1 or 200 ng/ml TNF and incubated further for 24 hours. Cells were lysed with 200 µl of 2× SDS sampling buffer containing 5% 2‐mercaptoethanol, and then the samples were analyzed by SDS‐PAGE under reducing conditions and Western blotting using anti–ADAM‐10, anti–ADAM‐17, anti–MMP‐14, and antiactin antibodies. Immune signals of each enzyme and actin were quantified using ImageJ software, and the relative amount of each enzyme was estimated using actin as an internal control.

### Analysis of the effect of inhibitory antibodies against ADAM‐17 and MMP‐14 on LRP‐1 protein levels

Normal chondrocytes (5 × 10^4^) cultured in 24‐well plates were rested in 500 μl of DMEM for 1 day. The medium was replaced with 500 μl of fresh DMEM with or without 10 ng/ml IL‐1 in the absence or presence of various concentrations of combinations of the control antibodies (human IgG for the anti–ADAM‐17 antibody [D1A12] and antidesmin intracellular domain antibody for the anti–MMP‐14 antibody [E2C6]) or the anti–ADAM‐17 and the anti–MMP‐14 antibodies. After 24 hours of incubation, the cells were lysed with 100 µl of 2× SDS sampling buffer, and then LRP‐1 α‐ and β‐chains were detected as described above. To test the effect of inhibitory antibodies against ADAM‐17 and MMP‐14 on LRP‐1 levels in OA chondrocytes and cartilage in culture, cells and cartilage explants were cultured for 24 hours with DMEM in the absence or presence of combinations of 250 n*M* each of the control antibodies, the anti–ADAM‐17 antibody and the antidesmin antibody, the anti–MMP‐14 antibody and IgG, or the anti–ADAM‐17 and the anti–MMP‐14 antibodies. LRP‐1 α‐ and β‐chains were then detected as described above.

### Analysis of aggrecan degradation in OA cartilage

OA cartilage was cultured in a round‐bottomed 96‐well plate (1 explant per well) and rested in DMEM for 1 day. The cartilage was further cultured in DMEM in the absence or presence of a combination of 2 antibodies (250 n*M* each) (i.e., combination of the anti–ADAM‐17 antibody and the antidesmin antibody [control], combination of the anti–MMP‐14 antibody and IgG [control], combination of the anti–ADAM‐17 and the anti–MMP‐14 antibodies, or combination of the antidesmin antibody and IgG), or 250 n*M* of anti–ADAMTS‐5 or N‐terminal domain of human TIMP‐3. After 12 hours of incubation, the medium was replaced with fresh DMEM containing the antibodies or TIMP and incubated further for 0–48 hours. For Western blotting, the conditioned media were pooled from each condition (3 explants per condition) and deglycosylated as described above, and immunoreactivity was measured within the linear range of the assay based on standard samples (see Supplementary Figure 1E, http://onlinelibrary.wiley.com/doi/10.1002/art.40080/abstract).

### Analysis of collagen degradation in OA cartilage

OA cartilage was cultured with various antibodies or with 250 n*M* of TIMP‐1 as described above for aggrecan degradation studies, and media were harvested after 96 hours. The extent of type II collagen degradation in OA cartilage was assessed by measuring the amount of hydroxyproline released into the media using a modification of the assay described by Bergman and Loxley 24. The hydroxyproline contents in cartilage explant remnants after culture were also determined by digesting them in 500 µl of papain digest solution (0.05*M* phosphate buffer [pH 6.5]/2 m*M N*‐acetylcysteine/2 m*M* EDTA/10 µg/ml papain) at 65°C for 24 hours. The relative amount of collagen degradation was estimated by dividing the amount of hydroxyproline released into the medium by the summed amount of hydroxyproline in the medium and in the papain digests.

### Study approval

Normal human articular cartilage tissue specimens were obtained from the Stanmore BioBank, Institute of Orthopaedics, Royal National Orthopaedic Hospital, Stanmore, following informed consent from patients and approval by the Royal Veterinary College Ethics and Welfare Committee (Institutional approval Unique Reference Number 2012 0048H). Human OA cartilage tissue specimens were obtained from the Oxford Musculoskeletal Biobank and were collected with informed donor consent in full compliance with national and institutional ethical requirements, the United Kingdom Human Tissue Act, and the Declaration of Helsinki (Human Tissue Authority Licence 12217 and Oxford Research Ethics Committee C 09/H0606/11).

### Statistical analysis

All quantified data are represented as the mean ± SD where applicable. Significant differences between data sets were determined using Student's 2‐tailed *t*‐test or one‐way analysis of variance followed by Dunnett's multiple comparison test, where indicated.

## RESULTS

### Increased ectodomain shedding of LRP‐1 and reduced endocytic capacity in human OA cartilage

**Figure 1:**
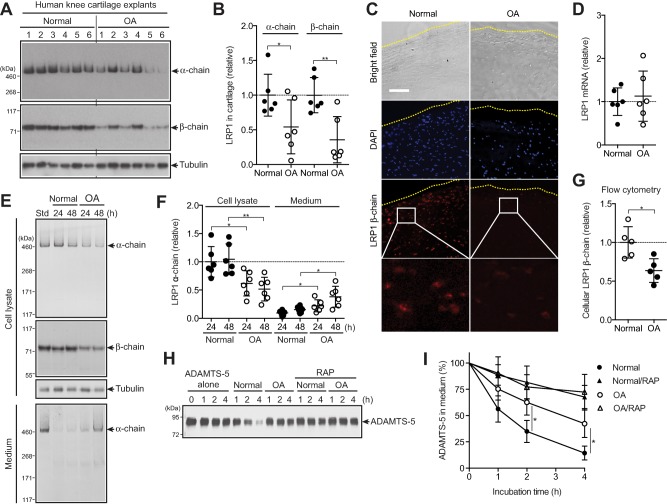
Increased ectodomain shedding of low‐density lipoprotein receptor–related protein 1 (LRP‐1) and reduced endocytic capacity of chondrocytes in human osteoarthritic (OA) cartilage. **A,** Western blotting of total proteins extracted from cartilage explants of human knee joints of OA patients and patients without arthritis (n = 6 each) with antibodies against α‐ and β‐chains of LRP‐1. **B,** Densitometric analysis of LRP‐1 in **A**. Data were normalized against tubulin. **C,** Immunofluorescence staining of LRP‐1 in frozen section of human knee cartilage. Dotted lines indicate the articular cartilage surface. Bar = 100 µm. **D,** Relative levels of mRNA for LRP‐1 in cartilage, measured by TaqMan quantitative reverse transcriptase–polymerase chain reaction. **E,** Representative Western blotting of LRP‐1 protein in cell lysates and medium of human normal and OA chondrocytes (n = 6 donors each). Std = standard cell lysates. **F,** Quantification of LRP‐1 α‐chain detected in **E**. **G,** Flow cytometric analysis of LRP‐1 β‐chain. **H,** Representative Western blotting for endocytosis of ADAMTS‐5 (10 n*M*) by human normal and OA chondrocytes (n = 3 donors each) with or without the LRP‐1 ligand antagonist receptor‐associated protein (RAP) (500 n*M*). ADAMTS‐5 in the medium was detected by Western blotting using anti–ADAMTS‐5 antibody. **I,** Quantification of findings in **H**. In **B**, **D**, **F**, and **G**, symbols represent individual cartilage donors; bars show the mean ± SD. The mean values in normal cartilage and chondrocytes were set at 1 (dashed lines). In **I**, values are the mean ± SD. ∗ = *P* < 0.05; ∗∗ = *P* < 0.01, by Student's 2‐tailed *t*‐test.

We first verified the loss of LRP‐1 protein in human OA cartilage. LRP‐1 consists of an extracellular 515‐kd α‐chain and an 85‐kd β‐chain that are processed from the precursor by furin. The α‐chain contains the ligand‐binding domains, and the β‐chain has an extracellular domain, a transmembrane domain, and a cytoplasmic domain. Western blotting analyses of α‐ and β‐chains of cartilage extracts with antibodies that recognize the N‐terminus of the α‐chain and the extracellular domain of the β‐chain showed that both chains were reduced in OA cartilage by ∼48% and ∼65%, respectively, compared with normal cartilage (Figures 1A and B). The reduction of LRP‐1 was further confirmed by immunofluorescence staining of β‐chain in the cartilage (Figure 1C). No significant change in the level of mRNA for LRP‐1 between normal and OA cartilage (Figure 1D) suggested that the loss of LRP‐1 in OA cartilage was due to proteolytic shedding of the receptor.

To further investigate the increased shedding of LRP‐1 in OA cartilage, chondrocytes were cultured and LRP‐1 proteins were analyzed. Both α‐ and β‐chains were reduced in OA cell lysates compared with normal chondrocytes, which was accompanied by an increased release of full‐length α‐chain into the medium (Figures 1E and F). Flow cytometric analysis of the β‐chain with antiectodomain antibody further confirmed the reduction of cell surface LRP‐1 including the β‐chain in OA chondrocytes (Figure 1G), suggesting that the primary shedding site is located in the ectodomain of the β‐chain. We estimated that a single normal chondrocyte released ∼3.9 ± 2.7 × 10^3^ LRP‐1 molecules per hour (mean ± SD), while a single OA chondrocyte released 9.6 ± 5.4 × 10^3^ LRP‐1 molecules per hour. As anticipated, the endocytic capacity of human OA chondrocytes was significantly reduced; the half‐life of ADAMTS‐5 was ∼2.8‐fold longer in OA chondrocytes (∼210 minutes) than in normal chondrocytes (∼75 minutes) (Figures 1H and I).

### Soluble LRP‐1 ectodomain prevents endocytosis of ADAMTS‐5 and MMP‐13 without interfering with their activities

**Figure 2:**
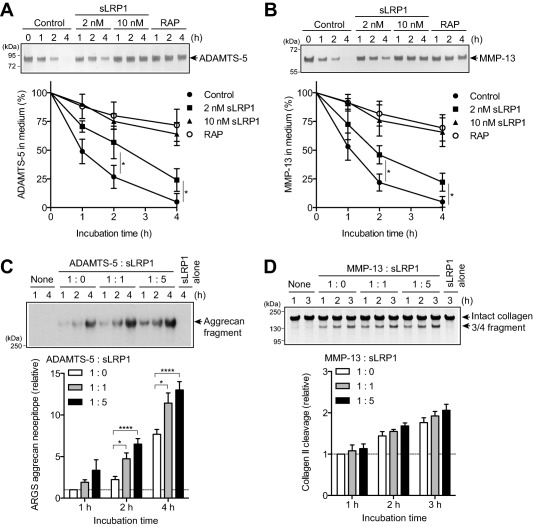
Ectodomain of soluble low‐density lipoprotein receptor–related protein 1 (sLRP‐1) prevents endocytosis of ADAMTS‐5 and matrix metalloproteinase 13 (MMP‐13) without interfering with their activities. **A** and **B,** Normal human chondrocytes (n = 3 donors) were cultured with Dulbecco's modified Eagle's medium containing 10 n*M* ADAMTS‐5 (**A**) or 10 n*M* MMP‐13 (**B**) with no additional treatment (Control) or in the presence of 2 n*M* sLRP‐1, 10 n*M* sLRP‐1, or 500 n*M* receptor‐associated protein (RAP) for 0–4 hours. ADAMTS‐5 and MMP‐13 in the medium were detected by Western blotting using anti–FLAG M2 antibody. Top, Representative Western blotting. Bottom, Quantification of findings in Western blotting. **C,** Bovine aggrecan (0.5 mg/ml) was incubated with 0.05 n*M* ADAMTS‐5 alone (1:0) or in the presence of 0.05 n*M* sLRP‐1 (1:1) or 0.25 n*M* sLRP‐1 (1:5) for 1–4 hours at 37°C. The reactions were stopped with 10 m*M* EDTA, and the reaction products were deglycosylated and subjected to Western blotting using antibody against the aggrecan neoepitope ^374^ARGSV (ARGS). Top, Representative Western blotting. Bottom, Quantification of findings in Western blotting. **D,** Type II collagen (1 mg/ml) was incubated with 5 n*M* MMP‐13 alone (1:0) or in the presence of 5 n*M* sLRP‐1 (1:1) or 25 n*M* sLRP‐1 (1:5) for 1–3 hours at 25°C. The reactions were stopped with 10 m*M* EDTA, and the reaction products were analyzed by sodium dodecyl sulfate–polyacrylamide gel electrophoresis (SDS‐PAGE) with Coomassie brilliant blue staining. Top, Representative SDS‐PAGE. Bottom, Quantification of findings in SDS‐PAGE. In **C** and **D**, the mean values after 1 hour of incubation without sLRP‐1 were set at 1 (dashed lines). Values are the mean ± SD. ∗ = *P* < 0.05; ∗∗∗∗ = *P* < 0.0001, by Student's 2‐tailed *t*‐test (**A** and **B**) or one‐way analysis of variance followed by Dunnett's multiple comparison test (**C**).

We then evaluated whether sLRP‐1 alters half‐lives of cartilage‐degrading metalloproteinases. As shown in Figures 2A and B, endocytosis of ADAMTS‐5 and MMP‐13 was reduced partially with 2 n*M* sLRP‐1 and almost completely inhibited with 10 n*M* sLRP‐1 to the level that was attained with the LRP ligand antagonist RAP. We also found that sLRP‐1–bound ADAMTS‐5 and MMP‐13 retained activity against their natural substrates, aggrecan and collagen, respectively (Figures 2C and D). It is notable that ADAMTS‐5 bound to sLRP‐1 was ∼3‐fold more active on aggrecan cleavage compared with free ADAMTS‐5 (Figure 2C). Thus, shedding of the LRP‐1 ectodomain impairs the endocytic capacity of the cell not only by reducing the level of cell surface LRP‐1 but also by converting membrane‐anchored LRP‐1 into soluble decoy receptors, leaving excess matrix‐degrading proteinases extracellularly.

### ADAM‐17 and MMP‐14 are responsible for shedding LRP‐1 in human chondrocytes

**Figure 3:**
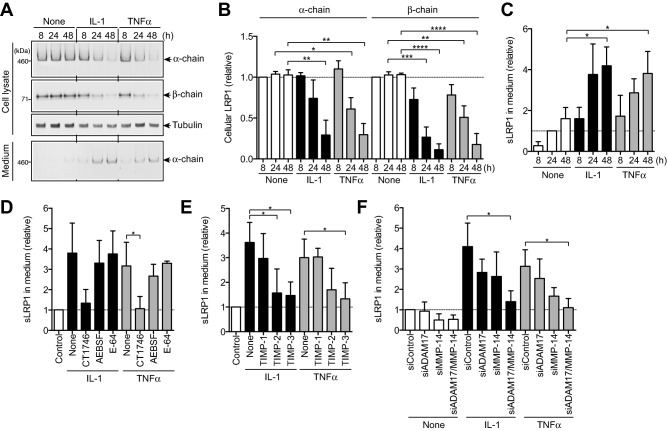
ADAM‐17 and matrix metalloproteinase 14 (MMP‐14) are the responsible sheddases of low‐density lipoprotein receptor–related protein 1 (LRP‐1) in human chondrocytes. **A,** Results of culturing human normal chondrocytes (n = 3 donors) with Dulbecco's modified Eagle's medium in the presence or absence of 10 ng/ml interleukin‐1 (IL‐1) or 200 ng/ml tumor necrosis factor (TNF) for 8–48 hours. LRP‐1 proteins in the cell lysate and the medium were analyzed by Western blotting with antibodies against α‐ and β‐chains of LRP‐1. **B** and **C,** Quantification of LRP‐1 protein in the cell lysate (**B**) and soluble LRP‐1 (sLRP‐1) released into the medium (**C**) with or without IL‐1 or TNF treatment. **D,** Effect of the metalloproteinase inhibitor CT1746 (20 µ*M*), the serine proteinase inhibitor 4‐(2‐aminoethyl)benzenesulfonyl fluoride (AEBSF) (50 µ*M*), or the cysteine proteinase inhibitor E‐64 (10 µ*M*) on cytokine‐induced LRP‐1 shedding with or without IL‐1 or TNF treatment. **E,** Effect of tissue inhibitor of metalloproteinases 1 (TIMP‐1) (500 n*M*), TIMP‐2 (500 n*M*), or TIMP‐3 (300 n*M*) on cytokine‐induced LRP‐1 shedding with or without IL‐1 or TNF treatment. **F,** Effect of small interfering RNA (siRNA)–mediated knockdown of ADAM‐17 and/or MMP‐14 on cytokine‐induced LRP‐1 shedding with or without IL‐1 or TNF treatment. Values in **B**–**F** are the mean ± SD. In **B**–**D**, the mean values in the cells incubated without cytokine for 8 hours (**B**) or 24 hours (**C** and **D**) were set at 1 (dashed lines). In **E** and **F**, the mean values in the cells transfected with nontargeting siRNA were set at 1 (dashed lines). ∗ = *P* < 0.05; ∗∗ = *P* < 0.002; ∗∗∗ = *P* < 0.0002; ∗∗∗∗ = *P* < 0.0001, by one‐way analysis of variance followed by Dunnett's multiple comparison test.

To identify the LRP‐1 sheddase in human chondrocytes, we first examined whether proinflammatory cytokines such as IL‐1 and TNF that stimulate cartilage matrix degradation increase LRP‐1 shedding, as this might facilitate characterization of the sheddase in the cartilage. As shown in Figures 3A–C, these cytokines increased LRP‐1 shedding ∼4.0‐fold in normal human chondrocytes. The cytokine‐stimulated LRP‐1 shedding was inhibited by the hydroxamate metalloproteinases inhibitor CT1746, but not by a serine proteinase inhibitor (AEBSF) or a cysteine proteinase inhibitor (E‐64) (Figure 3D). Among the 3 TIMPs tested, TIMP‐1 was not effective but TIMP‐2 and TIMP‐3 were, and TIMP‐3 showed the strongest inhibition (Figure 3E). Thus, we postulated that the responsible enzyme was likely to be a membrane‐anchored ADAM or MMP.

ADAM‐10, ADAM‐12, ADAM‐17, and MMP‐14 have previously been reported to be LRP‐1 sheddases in other cell types 25. We therefore ablated each enzyme individually using a specific siRNA. The ADAM‐10 protein level was reduced by ∼90%, and the ADAM‐12 mRNA level was reduced by ∼88% (see Supplementary Figures 2A–C, http://onlinelibrary.wiley.com/doi/10.1002/art.40080/abstract), but their knockdown did not affect LRP‐1 shedding (see Supplementary Figure 2D). Small interfering RNAs targeting ADAM‐17 and MMP‐14 reduced their protein levels by 76% and 84%, respectively (see Supplementary Figures 2E and F), and knockdown of each partially inhibited LRP‐1 shedding (Figure 3F). However, knockdown of both MMP‐14 and ADAM‐17 exhibited a stronger, additive effect, to the level achieved by TIMP‐3 (Figure 3F), which suggests that these 2 proteinases function as LRP‐1 sheddases. A low level of LRP‐1 shedding occurred in unstimulated chondrocytes, and was mainly due to MMP‐14.

### Combination of inhibitory antibodies against ADAM‐17 and MMP‐14 blocks LRP‐1 shedding in OA cartilage

**Figure 4:**
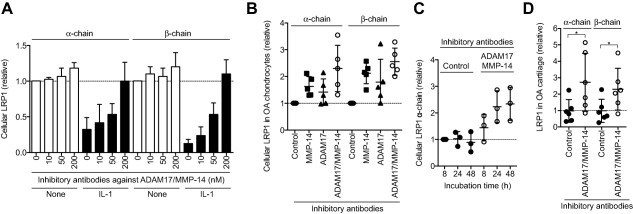
Combination of antibodies inhibiting ADAM‐17 and matrix metalloproteinase 14 (MMP‐14) blocks shedding of low‐density lipoprotein receptor–related protein 1 (LRP‐1) in osteoarthritic (OA) cartilage. **A,** Results of culturing human normal chondrocytes (n = 3 donors) with Dulbecco's modified Eagle's medium without (None) or with 10 ng/ml interleukin‐1 (IL‐1) in the absence or presence of combined anti–ADAM‐17 and anti–MMP‐14 (10–200 n*M* each) for 48 hours. LRP‐1 proteins in the cell lysate were analyzed by Western blotting with antibodies against α‐ and β‐chains of LRP‐1. **B,** Effect of combining anti–MMP‐14 and anti–ADAM‐17 antibodies (200 n*M* each) on LRP‐1 shedding in human OA chondrocytes (n = 5 donors). **C,** Time course analysis of the effect of combined anti–MMP‐14 and anti–ADAM‐17 antibodies on the recovery of cellular LRP‐1. **D,** Effect of combined anti–MMP‐14 and anti–ADAM‐17 antibodies on LRP‐1 shedding in human knee OA cartilage explants (n = 6 donors each). In **A**, values are the mean ± SD, and the mean value in the cells incubated without IL‐1 or the antibodies was set at 1 (dashed line). In **B**–**D**, symbols represent individual cartilage donors; bars show the mean ± SD. In **B**–**D**, the mean values in the cells or cartilage incubated with the control antibodies for 8 hours (**C**) or 24 hours (**B** and **D**) were set at 1 (dashed lines). ∗ = *P* < 0.05, by Student's 2‐tailed *t*‐test.

To verify the role of ADAM‐17 and MMP‐14 in LRP‐1 shedding, we used the recently developed specific antibodies against ADAM‐17 (D1A12) 15 and against MMP‐14 (E2C6) 16, which inhibit the target enzymes with inhibition constants of 0.46 n*M* and 0.11 n*M*, respectively. The IL‐1–induced loss of LRP‐1 was partially inhibited by a single antibody, but a combination of the 2 antibodies blocked LRP‐1 shedding to the level of IL‐1–untreated cells (Figure 4A). They were similarly effective at blocking LRP‐1 shedding in OA chondrocytes (Figure 4B). The anti–MMP‐14 antibody increased cellular levels of the LRP‐1 α‐ and β‐chains 1.6‐fold and 2.1‐fold, respectively, while the anti–ADAM‐17 antibody increased them 1.4‐fold and 1.8‐fold, respectively. A combination of the 2 antibodies increased both α‐ and β‐chains 2.3‐fold and 2.6‐fold, respectively (Figure 4B). The restoration of LRP‐1 in OA chondrocytes by combining the 2 antibodies was time dependent, and it reached a plateau at 24 hours (Figure 4C). Addition of the 2 antibodies to OA cartilage blocked LRP‐1 shedding and increased α‐ and β‐chains 2.6‐fold and 2.3‐fold, respectively (Figure 4D). This indicates that the antibodies can penetrate the tissue and inhibit the LRP‐1 sheddases in OA cartilage.

### Blocking of LRP‐1 sheddases restores endocytic capacity and reduces the degradation of aggrecan and collagen in OA cartilage

**Figure 5:**
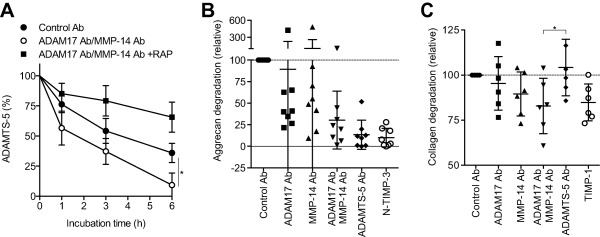
Blocking of low‐density lipoprotein receptor–related protein 1 (LRP‐1) sheddases restores endocytic capacity and reduces aggrecan and collagen degradation in osteoarthritic (OA) cartilage. **A,** Recovery of endocytic capacity in OA chondrocytes upon treatment with anti–ADAM‐17 and anti–matrix metalloproteinase 14 (anti–MMP‐14) antibodies (Ab). Endocytosis of ADAMTS‐5 (10 n*M*) was measured as described in Figures 1H and I (n = 3 donors). **B** and **C,** Inhibition of aggrecan and collagen degradation in human OA cartilage upon treatment with combined anti–ADAM‐17 and anti–MMP‐14 antibodies. OA knee cartilage explants were incubated with antibodies or tissue inhibitor of metalloproteinases (TIMP) at 250 n*M*. **B,** Aggrecan degradation detected after 48 hours with an antibody against the aggrecan neoepitope ^374^ARGSV. The mean value in medium of the cartilage incubated with the control antibodies was set at 100 (dashed line) (n = 8 donors). **C,** Collagen degradation of cultured OA cartilage after 96 hours of incubation, measured by hydroxyproline assay. The amount of hydroxyproline released with control antibodies was set at 100 (dashed line). In **A**, values are the mean ± SD. In **B** and **C**, symbols represent individual cartilage donors; bars show the mean ± SD. The mean values in media of the cartilage incubated with the control antibodies for 48 hours (**B**) or 96 hours (**C**) were set at 100 (dashed lines). ∗ = *P* < 0.05, by Student's 2‐tailed *t*‐test (**A**) or one‐way analysis of variance followed by Dunnett's multiple comparison test (**C**). RAP = receptor‐associated protein; N‐TIMP‐3 = N‐terminal domain of human TIMP‐3.

We then tested the effect of combining the 2 antibodies on the endocytic capacity of OA chondrocytes. The antibody‐treated OA chondrocytes cleared exogenously added ADAMTS‐5 from the medium ∼2.4‐fold faster (half‐life ∼95 minutes) than the untreated OA chondrocytes (half‐life ∼210 minutes) (Figure 5A), indicating that blocking LRP‐1 sheddases restored the endocytic capacity of OA chondrocytes close to that of normal chondrocytes.

Remarkably, combined antibody treatment reduced the degradation of aggrecan and collagen in OA cartilage. Analysis of the same conditioned media for the aggrecanase‐specific cleavage motif using the antibody against the ^374^ARGSV neoepitope indicated that aggrecanase activity was markedly inhibited by blocking LRP‐1 sheddases (Figure 5B) (see Supplementary Figure 3, http://onlinelibrary.wiley.com/doi/10.1002/art.40080/abstract). Potent inhibition with the anti–ADAMTS‐5 antibody and TIMP‐3 indicated that the primary aggrecanase in OA cartilage was ADAMTS‐5. Effective inhibition of aggrecan degradation by the combination of anti–ADAM‐17 and anti–MMP‐14 antibodies was further confirmed by Safranin O staining of the cartilage (see Supplementary Figure 4, http://onlinelibrary.wiley.com/doi/10.1002/art.40080/abstract).

Collagen degradation was also inhibited in the presence of anti–ADAM‐17 and anti–MMP‐14 antibodies by 5% and 10%, respectively, and more effective inhibition (17%) was observed upon combining the 2 antibodies (Figure 5C). In general, anti–MMP‐14 showed a stronger effect than anti–ADAM‐17, but there was considerable patient‐to‐patient variation in the effect of each antibody, which may reflect the multifactorial nature of OA. Inhibition was also detected with TIMP‐1 treatment, but not with the anti–ADAMTS‐5 antibody, indicating that collagen degradation is specific to collagenase. Cell viability analysis indicated that none of these treatments was toxic to chondrocytes (data not shown).

## DISCUSSION

In this study, we have shown that the ectodomain shedding of LRP‐1 may be an important regulator of the development of human OA. The specific inhibitory antibodies that we recently developed for human MMP‐14 and ADAM‐17 have allowed us to evaluate the role of LRP‐1 shedding in degradation of cartilage matrix in human subjects and thus provided clinically relevant information.

Numerous membrane‐anchored proteins are released from the cell surface by the process of regulated proteolysis called ectodomain shedding, and the enzymes responsible for shedding are primarily membrane‐anchored proteinases. This process regulates a wide variety of cellular and physiologic functions, and dysregulated shedding is linked to numerous diseases, such as Alzheimer's disease, inflammation, rheumatoid arthritis (RA), cancer, chronic kidney disease, cardiac hypertrophy, and heart failure 26, 27. LRP‐1 shedding is increased under inflammatory conditions such as in RA and systemic lupus erythematosus 28, and in cancer 29, 30, but the exact pathologic role of LRP‐1 shedding in these diseases has not been clearly understood. We propose that LRP‐1 shedding in local tissues under inflammatory or chronic pathologic conditions dysregulates normal turnover of ECM and cellular homeostasis, leading to slowly progressing chronic diseases such as in OA.

LRP‐1 is widely expressed in different cell types and controls extracellular levels of numerous biologically active molecules to maintain tissue homeostasis 31. Currently, more than 50 ligands have been characterized, including lipoproteins, ECM proteins, growth factors, cell surface receptors, proteinases, proteinase inhibitors, and secreted intracellular proteins 31. In cartilage, LRP‐1 controls not only ECM‐degrading proteinases but also the Wnt/β‐catenin signaling pathway by interacting with Frizzled‐1 32 and connective tissue growth factor (CCN2), and both regulate endochondral ossification and articular cartilage regeneration 33, emphasizing the importance of LRP‐1 in skeletal development and in the maintenance of cartilage homeostasis.

Thus, the impairment of LRP‐1 function due to increased shedding of the receptor is detrimental to healthy cartilage, as demonstrated in the present study. This is triggered by increased activity of ADAM‐17 and MMP‐14, but their protein levels were not significantly changed between healthy and OA cartilage (data not shown), which suggests that the activation of these enzymes is regulated posttranslationally. The additive but not synergistic effect of anti–ADAM‐17 and anti–MMP‐14 antibodies further suggests that these proteinases may be activated by different mechanisms and act independently as LRP‐1 sheddases. In addition, MMP‐14 and ADAM‐17 cleave a number of cell membrane proteins, including growth factors, cytokines, cell adhesion molecules, and mechanosensors 26, 34. Therefore, their activation may also affect the integrity of other cell surface molecules in cartilage as well as cellular behavior. We are currently investigating how ADAM‐17 and MMP‐14 are activated as well as their substrate selectivity in cartilage, as these may indicate additional molecular mechanisms for the development of OA, particularly in the early stages.

**Figure 6:**
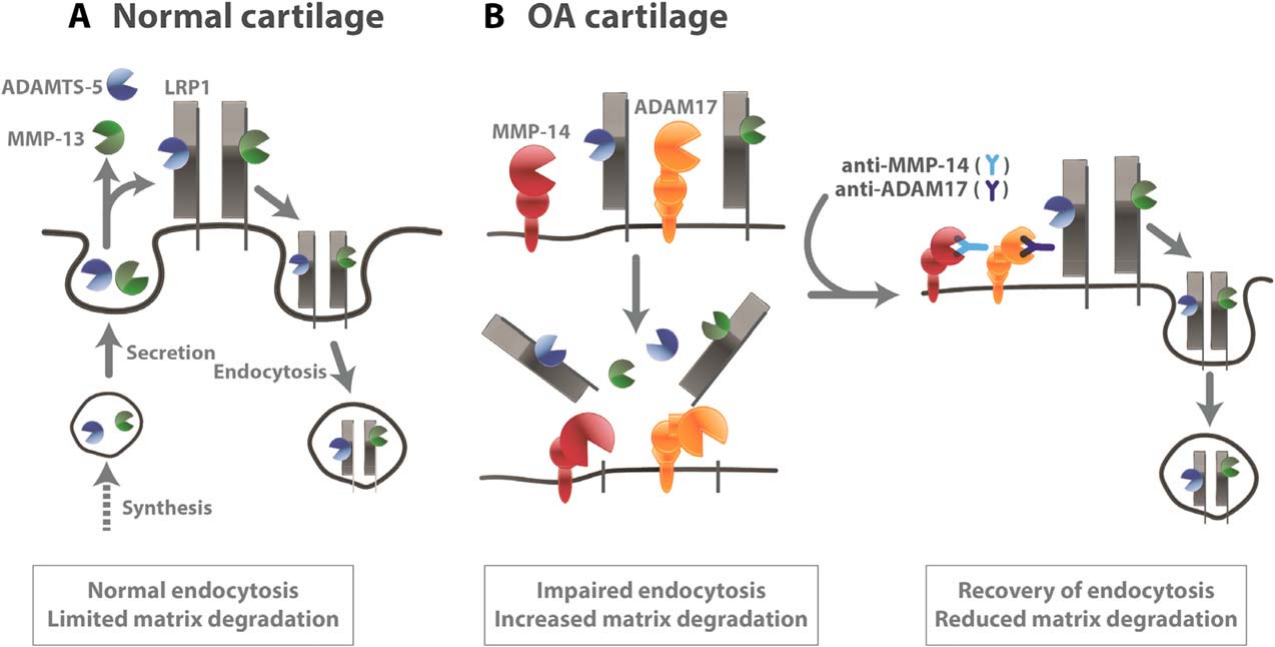
Low‐density lipoprotein receptor–related protein 1 (LRP‐1)–mediated endocytic pathways in normal and osteoarthritic (OA) cartilage. **A,** Secreted ADAMTS‐5 and matrix metalloproteinase 13 (MMP‐13) are largely endocytosed by LRP‐1 in normal cartilage. Thus, their activities in the extracellular milieu are limited. **B,** In OA cartilage, the ectodomain of LRP‐1 is shed by ADAM‐17 and MMP‐14, resulting in impairment of endocytic capacity of chondrocytes. Blocking of ADAM‐17 and MMP‐14 activities recovers the lost endocytic function of OA chondrocytes, reduces cartilage matrix degradation, and restores cartilage homeostasis.

Another notable finding of this study is that anti–ADAM‐17 and anti–MMP‐14 antibodies reduced both aggrecanolytic and collagenolytic activities of human OA cartilage in culture, and this effect was due to restoration of the lost LRP‐1 function by blocking the LRP‐1 sheddase activities of ADAM‐17 and MMP‐14 (Figure 6). These results suggest that inhibition of elevated LRP‐1 sheddase activities in OA cartilage may be an effective way to prevent cartilage matrix degradation. Although the systematic inhibition of ADAM‐17 and MMP‐14 as OA therapy may be problematic, as these enzymes are biologically important in the release of growth factors and cell surface receptors in many cell types 27, 34, local administration of anti–ADAM‐17 and anti–MMP‐14 antibodies or small molecule inhibitors of ADAM‐17 and MMP‐14 may be worth investigating as disease‐modifying OA drugs. This approach is an attractive option for OA therapy, as the recovery of the lost endocytic function of chondrocytes would help to maintain cartilage homeostasis. We are currently testing whether this approach is beneficial in early and advanced OA, using preclinical animal models.

## AUTHOR CONTRIBUTIONS

All authors were involved in drafting the article or revising it critically for important intellectual content, and all authors approved the final version to be published. Dr. Yamamoto had full access to all of the data in the study and takes responsibility for the integrity of the data and the accuracy of the data analysis.

### Study conception and design

Yamamoto, Murphy, Nagase.

### Acquisition of data

Yamamoto, Santamaria, Botkjaer, Dudhia.

### Analysis and interpretation of data

Yamamoto, Troeberg, Itoh, Murphy, Nagase.

## Supporting information

Supplementary Figure 1. Verification for a linear relationship between mass of antigen and Western blotting signals for LRP1 α‐ and β‐chains, ADAMTS‐5, MMP‐13 and ARGS aggrecan fragments. The different amounts of the cell lysates of human chondrocytes used in Fig. 1E (A and B), the pre‐incubation (0‐h) samples used in Fig. 1H and Fig. 2B, and the deglycosylated conditioned medium used in Fig. 5B and Fig. S2 were analysed by Western blotting using anti‐LRP1 α‐chain (A), anti‐LRP1 β‐chain (B), anti‐ADAMTS‐5 catalytic domain (C), anti‐FLAG M2 (D), and anti‐^374^ARGS aggrecan neoepitope (E) antibodies, respectively. The immune signals of each sample were quantified using ImageJ.
Supplementary Figure 2. siRNA‐mediated knockdown of ADAM10, ADAM12, ADAM17 and MMP‐14 in human chondrocytes. (A and B) Human normal chondrocytes (n=3) transfected with non‐targeting siRNA, siRNA targeting ADAM10, ADAM17 or MMP‐14 were cultured for 2 days in DMEM. The media were removed and fresh DMEM without or with 10 ng/ml IL‐1 or 200 ng/ml TNFα was added, and the cells were further cultured for 24 h. Each metalloproteinase in the cell lysates was detected by Western blot analysis using antibodies specific for each metalloproteinase. (A) Representative Western blot analysis. (B) The immune signals of ADAM10 were quantified using ImageJ and normalized using actin as an internal control, where the mean value for each proteinase in the cells transfected with non‐targeting siRNA without cytokine treatment was taken as 100. (C) Human normal chondrocytes (n=3) transfected with non‐targeting siRNA or siRNA targeting ADAM12 were cultured for 2 days in DMEM. The media were removed and fresh DMEM without or with 10 ng/ml IL‐1 or 200 ng/ml TNFα was added, and the cells were further cultured for 24 h. Total mRNA was extracted from the cells and relative ADAM12 mRNA levels are measured using TaqMan qPCR analysis. (D) Effect of siRNA‐mediated knockdown of ADAM10 or ADAM12 on cytokine‐induced LRP1 shedding. The mean value of the cells transfected with non‐targeting siRNA was taken as 1. (E and F) Human normal chondrocytes (n=3) were transfected with non‐targeting siRNA, siRNA targeting ADAM17 or MMP‐14, or the combination of siRNAs targeting ADAM17 and MMP‐14 for 2 days in DMEM. The media were removed and fresh DMEM without or with IL‐1 or TNFα was added, and the cells were further cultured for 24 h. ADAM17 (E) and MMP‐14 (F) in the cell lysates were detected and quantified as in A. Data are expressed as the mean ± SD.
Supplementary Figure 3. Effect of the inhibitory antibodies against ADAM17 and MMP‐14 on the degradation of aggrecan in human OA cartilage.
The cartilage explants were dissected from the knee joints of human OA patients (n=8) and rested with DMEM for 2 days. The cartilage was further incubated with combinations of the control antibodies, the anti‐ADAM17 antibody and the control antibody, the anti‐MMP‐14 antibody and the control antibody, or the anti‐ADAM17 and the anti‐MMP‐14 antibodies, or the inhibitory antibody against ADAMTS‐5 (2D3), or N‐TIMP‐3 (each 250 nM). After 12 h incubation, the medium was replaced with fresh DMEM containing the antibodies or TIMPs and further incubated for 48 h. The conditioned medium was then deglycosylated and subjected to Western blot analysis for aggrecan fragments using an anti‐ARGS neoepitope antibody. yr; years‐old, M; male, F; female.
Supplementary Figure 4. Safranin O staining of human OA cartilage treated with the inhibitory antibodies against ADAM17 and MMP‐14.
The cartilage explants were dissected from the knee joints of human OA patients (n=3) and rested with DMEM for 2 days. A cartilage piece was halved and one piece was further incubated with combinations of the anti‐ADAM17 and the anti‐MMP‐14 antibodies, or control antibodies (anti‐Desmin and human IgG) for 96 h. Two paired pieces of cartilage were then fixed with formalin, put together and embedded in paraffin wax, and sectioned (5‐µm sections) for Safranin O staining. Dashed line, articular cartilage surface. Scale bar, 1 mm.
