# Supplementary material for: Inhibition of Shedding of Low‐Density Lipoprotein Receptor–Related Protein 1 Reverses Cartilage Matrix Degradation in Osteoarthritis
Source: Arthritis Rheumatol. 2017 Apr 28;69(6):1246–56. doi: 10.1002/art.40080 (PMC5449214; doi:10.1002/art.40080)
Supplement: Supplementary file 1 — Supplementary Figure 1. Verification for a linear relationship between mass of antigen and Western blotting signals for LRP1 α‐ and β‐chains, ADAMTS‐5, MMP‐13 and ARGS aggrecan fragments. The different amounts of the cell lysates of human chondrocytes used in Fig. 1E (A and B), the pre‐incubation (0‐h) samples used in Fig. 1H and Fig. 2B, and the deglycosylated conditioned medium used in Fig. 5B and Fig. S2 were analysed by Western blotting using anti‐LRP1 α‐chain (A), anti‐LRP1 β‐chain (B), anti‐ADAMTS‐5 catalytic domain (C), anti‐FLAG M2 (D), and anti‐374ARGS aggrecan neoepitope (E) antibodies, respectively. The immune signals of each sample were quantified using ImageJ. Supplementary Figure 2. siRNA‐mediated knockdown of ADAM10, ADAM12, ADAM17 and MMP‐14 in human chondrocytes. (A and B) Human normal chondrocytes (n=3) transfected with non‐targeting siRNA, siRNA targeting ADAM10, ADAM17 or MMP‐14 were cultured for 2 days in DMEM. The media were removed and fresh DMEM without or with 10 ng/ml IL‐1 or 200 ng/ml TNFα was added, and the cells were further cultured for 24 h. Each metalloproteinase in the cell lysates was detected by Western blot analysis using antibodies specific for each metalloproteinase. (A) Representative Western blot analysis. (B) The immune signals of ADAM10 were quantified using ImageJ and normalized using actin as an internal control, where the mean value for each proteinase in the cells transfected with non‐targeting siRNA without cytokine treatment was taken as 100. (C) Human normal chondrocytes (n=3) transfected with non‐targeting siRNA or siRNA targeting ADAM12 were cultured for 2 days in DMEM. The media were removed and fresh DMEM without or with 10 ng/ml IL‐1 or 200 ng/ml TNFα was added, and the cells were further cultured for 24 h. Total mRNA was extracted from the cells and relative ADAM12 mRNA levels are measured using TaqMan qPCR analysis. (D) Effect of siRNA‐mediated knockdown of ADAM10 or ADAM12 on cytokine‐induced LRP1 shedding. Th [file ART-69-1246-s001.docx]

**ONLINE SUPPLEMENTARY FIGURES**

**
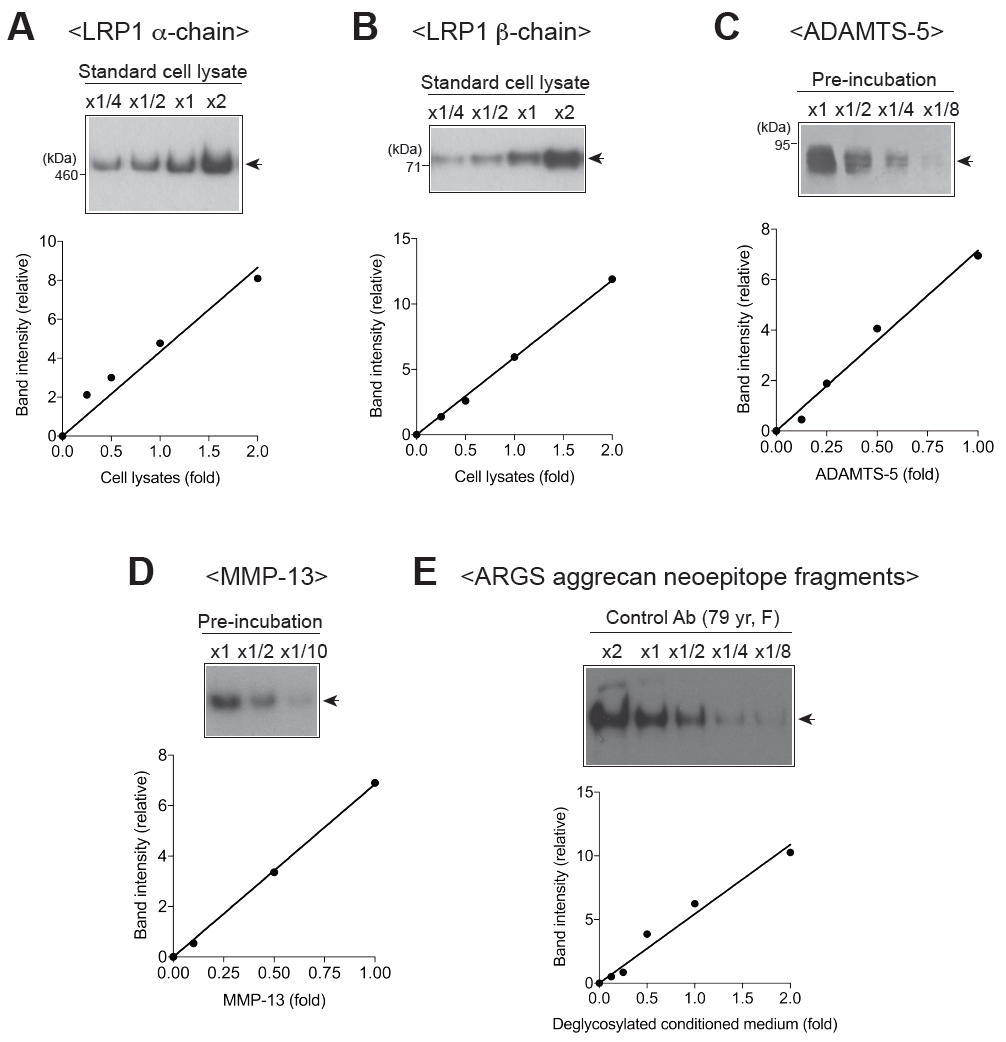
**

**Supplementary Figure 1. Verification for a linear relationship between mass of antigen and Western blotting signals for LRP1 α- and β-chains, ADAMTS-5, MMP-13 and ARGS aggrecan fragments.** The different amounts of the cell lysates of human chondrocytes used in **Fig. 1E** (**A** and **B**)**,** the pre-incubation (0-h) samples used in **Fig. 1H** and **Fig. 2B**, and the deglycosylated conditioned medium used in **Fig. 5B** and **Fig. S2** were analysed by Western blotting using anti-LRP1 α-chain (**A**), anti-LRP1 β-chain (**B**), anti-ADAMTS-5 catalytic domain (**C**), anti-FLAG M2 (**D**), and anti-^374^ARGS aggrecan neoepitope (**E**) antibodies, respectively. The immune signals of each sample were quantified using ImageJ.

**
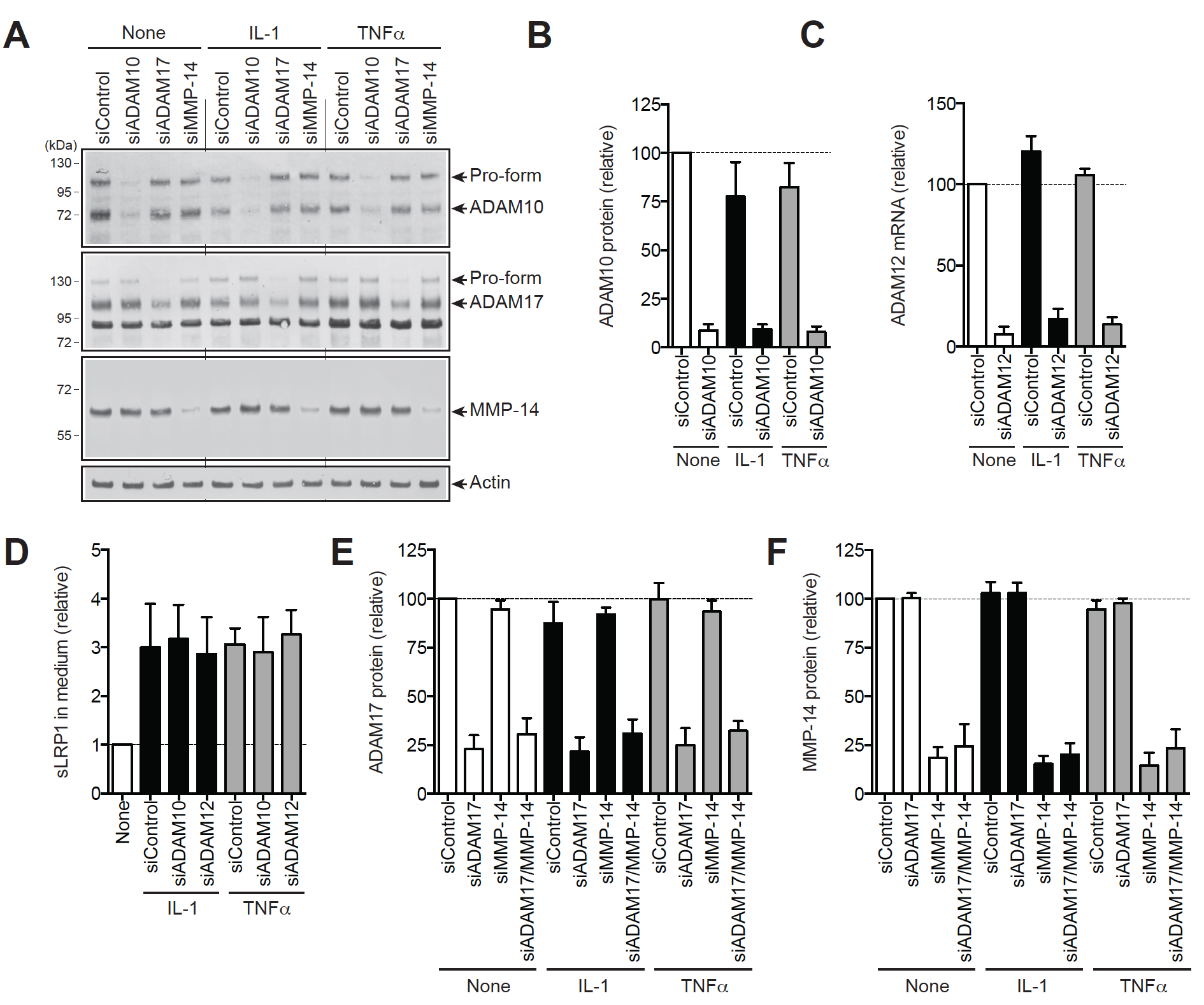
**

**Supplementary Figure 2. siRNA-mediated knockdown of ADAM10, ADAM12, ADAM17 and MMP-14 in human chondrocytes.** (**A** and **B**) Human normal chondrocytes (n=3) transfected with non-targeting siRNA, siRNA targeting ADAM10, ADAM17 or MMP-14 were cultured for 2 days in DMEM. The media were removed and fresh DMEM without or with 10 ng/ml IL-1 or 200 ng/ml TNFα was added, and the cells were further cultured for 24 h. Each metalloproteinase in the cell lysates was detected by Western blot analysis using antibodies specific for each metalloproteinase. (**A**) Representative Western blot analysis. (**B**) The immune signals of ADAM10 were quantified using ImageJ and normalized using actin as an internal control, where the mean value for each proteinase in the cells transfected with non-targeting siRNA without cytokine treatment was taken as 100. (**C**) Human normal chondrocytes (n=3) transfected with non-targeting siRNA or siRNA targeting ADAM12 were cultured for 2 days in DMEM. The media were removed and fresh DMEM without or with 10 ng/ml IL-1 or 200 ng/ml TNFα was added, and the cells were further cultured for 24 h. Total mRNA was extracted from the cells and relative ADAM12 mRNA levels are measured using TaqMan qPCR analysis. (**D**) Effect of siRNA-mediated knockdown of ADAM10 or ADAM12 on cytokine-induced LRP1 shedding. The mean value of the cells transfected with non-targeting siRNA was taken as 1. (**E** and **F**) Human normal chondrocytes (n=3) were transfected with non-targeting siRNA, siRNA targeting ADAM17 or MMP-14, or the combination of siRNAs targeting ADAM17 and MMP-14 for 2 days in DMEM. The media were removed and fresh DMEM without or with IL-1 or TNFα was added, and the cells were further cultured for 24 h. ADAM17 (**E**) and MMP-14 (**F**) in the cell lysates were detected and quantified as in **A**. Data are expressed as the mean ± SD.


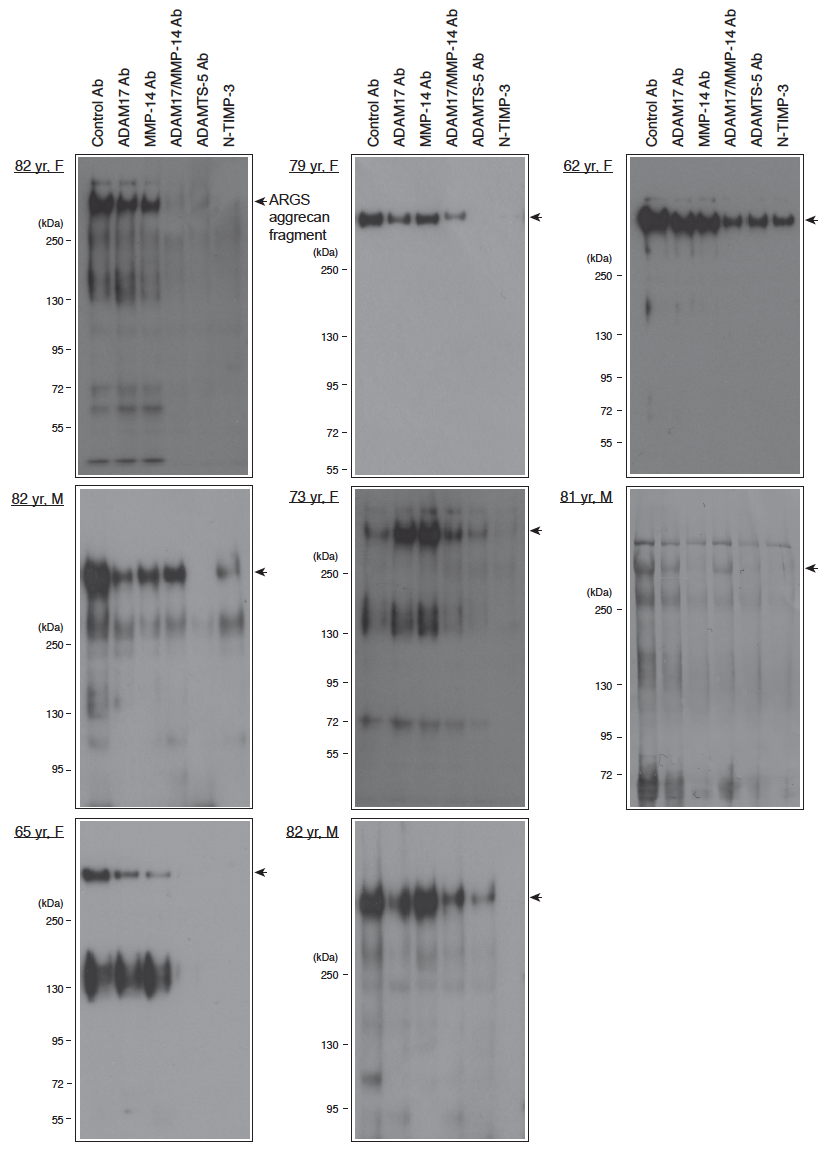


**Supplementary Figure 3. Effect of the inhibitory antibodies against ADAM17 and MMP-14 on the degradation of aggrecan in human OA cartilage.**

The cartilage explants were dissected from the knee joints of human OA patients (n=8) and rested with DMEM for 2 days. The cartilage was further incubated with combinations of the control antibodies, the anti-ADAM17 antibody and the control antibody, the anti-MMP-14 antibody and the control antibody, or the anti-ADAM17 and the anti-MMP-14 antibodies, or the inhibitory antibody against ADAMTS-5 (2D3), or N-TIMP-3 (each 250 nM). After 12 h incubation, the medium was replaced with fresh DMEM containing the antibodies or TIMPs and further incubated for 48 h. The conditioned medium was then deglycosylated and subjected to Western blot analysis for aggrecan fragments using an anti-ARGS neoepitope antibody. yr; years-old, M; male, F; female.

**
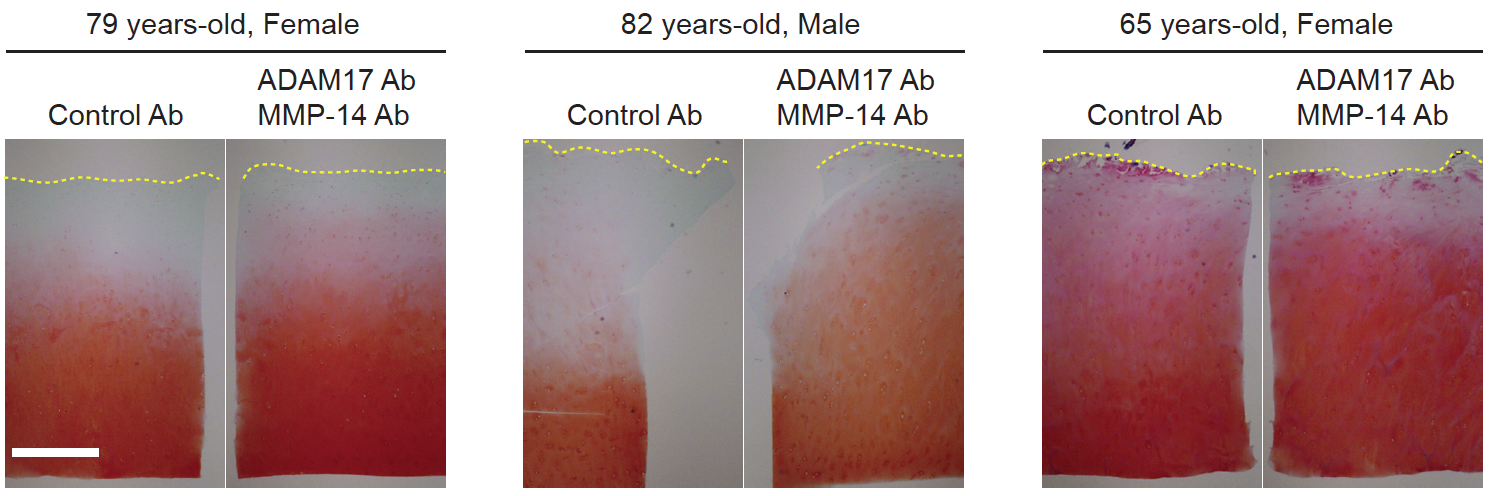
**

**Supplementary Figure 4. Safranin O staining of human OA cartilage treated with the inhibitory antibodies against ADAM17 and MMP-14.**

The cartilage explants were dissected from the knee joints of human OA patients (n=3) and rested with DMEM for 2 days. A cartilage piece was halved and one piece was further incubated with combinations of the anti-ADAM17 and the anti-MMP-14 antibodies, or control antibodies (anti-Desmin and human IgG) for 96 h. Two paired pieces of cartilage were then fixed with formalin, put together and embedded in paraffin wax, and sectioned (5-µm sections) for Safranin O staining. Dashed line, articular cartilage surface. Scale bar, 1 mm.
